# Incidence and Characterization of *Salmonella* Isolates From Raw Meat Products Sold at Small Markets in Hubei Province, China

**DOI:** 10.3389/fmicb.2019.02265

**Published:** 2019-10-04

**Authors:** Min Zhou, Xiaofang Li, Wenfu Hou, Hongxun Wang, George C. Paoli, Xianming Shi

**Affiliations:** ^1^School of Food Science and Engineering, Wuhan Polytechnic University, Wuhan, China; ^2^Molecular Characterization of Foodborne Pathogens Research Unit, United States Department of Agriculture, Agricultural Research Service, Eastern Regional Research Center (USDA-ARS-ERRC), USDA–MOST Joint Research Center for Food Safety, Wyndmoor, PA, United States; ^3^State Key Laboratory of Microbial Metabolism, School of Agriculture and Biology, MOST-USDA Joint Research Center for Food Safety, Shanghai Jiao Tong University, Shanghai, China

**Keywords:** *Salmonella*, MLST, class I integrons, *bla*_CTX–M–65_, *bla*_CTX–M–15_, *qnrB*

## Abstract

*Salmonella* is a leading cause of foodborne disease and is often associated with the consumption of foods of animal origin. In this study, sixty-six *Salmonella* isolates were obtained from 631 raw meat samples purchased at small retail suppliers in Hubei Province, China. The most prevalent *Salmonella* serotypes were Thompson (18.2%) and Agona (13.6%). Frequent antimicrobial resistance was observed for the sulfonamides (43.9%), tetracycline (43.9%), and the β-lactams amoxicillin and ampicillin (36.4% for each). Interestingly, a high incidence of resistance to cephazolin was observed in strains of the most common serotype, *S.* Thompson. Class I integrons were found in 27.3% (18/66) of the isolates and five of these integrons contained different gene cassettes (*aacA4C-arr-3-dfr2, dfrA12-aadA21, aadA2, dfrA12-aadA2, dfr17-aadA5*). Additional antimicrobial resistance genes, including *bla*_TEM–1_, *bla*_CTX–M–65_, *bla*_CTX–M–15_, *qnrB*, and *qnrS*, were also identified among these *Salmonella* isolates. Results of replicon typing and conjugation experiments revealed that an integron with *qnrB* and *bla*_CTX–M–15_ genes was present on incH12 mobile plasmid in *S.* Thompson strain. Multilocus sequence typing (MLST) analysis revealed 32 sequence types, indicating that these isolates were phenotypically and genetically diverse, among which ST26 (18.2%) and ST541 (12.1%) were the predominant sequence types. The integrons, along with multiple antimicrobial resistance genes on mobile plasmids, are likely contributors to the dissemination of multidrug resistance in *Salmonella*.

## Introduction

*Salmonella* is an important source of foodborne disease, resulting in morbidity and mortality worldwide (Park et al., 2015). In China, 70–80% of bacterial food poisoning cases are due to *Salmonella* that originates in poultry, eggs, beef and pork (Yang et al., 2013). Direct and indirect spread of *Salmonella* between animals and humans are threats to human health. Although more than 2610 serotypes of *Salmonella* have been identified, the majority of human *Salmonella* infections are caused by strains of only a few serotypes (Mancin et al., 2015). Therefore, serotype determination is an important aspect of epidemiological surveillance and disease assessment (Hung et al., 2017). Changes in the prevalence of specific serotypes can result from the movements of people, animals, and foods (Hendriksen et al., 2009). At present, serotypes Enteritidis and Typhimurium have been most frequently implicated in salmonellosis outbreaks from foods in Taiwan, Greece, Qatar and South Africa (Chang et al., 2016; Papadopoulos et al., 2016; Hung et al., 2017; Smith et al., 2017).

Though not as facile or rapid as serotyping, multilocus sequence typing (MLST) is a reliable and highly discriminatory molecular typing method. In this technique, the phylogenetic relationship between bacterial strains is determined based upon nucleotide sequence variations within several housekeeping and other conserved genes (Chang et al., 2016). MLST has many advantages compared with other genotyping techniques (Stepan et al., 2011). It is consistent and reproducible, and combines nucleotide sequencing and bioinformatics with a population genetics approach to produce accurate, reliable and highly portable results (Smith et al., 2017). Furthermore, it has been suggested that MLST is superior to serotyping for *Salmonella* surveillance and outbreak tracking (Achtman et al., 2012).

The widespread use of antibiotics poses problems for antimicrobial resistance, which leads to an increase in treatment costs and even to therapy failure (Hur et al., 2012). Multiple drug resistance (MDR) among *Salmonella* is prevalent. This applies not only to traditional antimicrobials such as sulfamethoxazole and chloramphenicol, but also against clinically important antimicrobials like the third generation β-lactams and fluoroquinolones (Agada et al., 2014). The selective pressure caused by the application of antimicrobials in poultry production and veterinary practice for growth promotion and prophylaxis has resulted in an increase in antibiotic resistance and an increase in the presence of genes conferring antimicrobial resistance to *Salmonella* (Zishiri et al., 2016). The spread of antimicrobial resistance in *Salmonella* is primarily due to integrons (Rajaei et al., 2014). Integrons are mobile DNA elements containing multiple antimicrobial resistant genes, which can move from one bacterium to another. This increases multi-drug resistance in bacterial populations (Asgharpour et al., 2014). Among various integrons, the class I integron is frequently observed among antimicrobial resistant *Salmonella* and plays a major role in the dissemination of resistance genes (Wright, 2010).

To the best of our knowledge, there is limited information regarding the incidence of *Salmonella* in retail raw meat products in Central China. Therefore, the objective of this study was to determine the prevalence, serotype distribution, genetic diversity, antimicrobial resistance, and the presence of class I integrons of *Salmonella* from retail raw meat products in Hubei Province, China. This data can be used for quantitative risk assessments of *Salmonella* isolates in retail meat and to help establish a series of prevention and control measures against foodborne pathogenic bacteria.

## Materials and Methods

### Sample Collection and *Salmonella* Isolation and Serotyping

We collected 631 retail raw meat samples, including 263 chicken meat, 102 duck meat, 80 pork and 186 fish products from 20 small markets, in Wuhan City of Hubei Province, China, from 2014 to 2017. All samples were transported to our laboratory on ice within 6 h of collection. *Salmonella* isolation and identification methods were carried out according to the standard procedures of National Food Safety Standard Food Microbiological Examination: *Salmonella* (GB 4789.4-2016). Biochemical identification was done using an HBI microbial biochemical identification kit (Hope Biotechnology, Qingdao, China).

Serotyping of *Salmonella* isolates was done according to the Kauffmann-White scheme using an O and H antigen slide agglutination kit (Tianrun Biopharmaceutical, Ningbo, China) (Zhao et al., 2017).

### Antimicrobial Susceptibility Testing

Antimicrobial susceptibility testing was performed using the Kirby-Bauer disk diffusion method as described by the Clinical and Laboratory Standards Institute (Clinical and Laboratory Standards Institute [CLSI], 2016). Antimicrobial disks (Oxoid Ltd., Basingstoke, United Kingdom) used for testing contained 10 μg amoxicillin, 10 μg ampicillin, 30 μg cefotaxime, 30 μg cefuroxime sodium, 30 μg cephazolin, 30 μg chloramphenicol, 5 μg ciprofloxacin, 30 μg nalidixic acid, 10 μg norfloxacin, 10 μg streptomycin, 10 μg gentamicin, 300 μg nitrofurantoin, 300 μg sulfonamide and 30 μg tetracycline. *Escherichia coli* strain ATCC 25922 was used as a reference strain. The standard break points were interpreted according to the guidelines of the Clinical and Laboratory Standards Institute (Clinical and Laboratory Standards Institute [CLSI], 2016).

### Antimicrobial Resistance Genes and Class I Integron Identification

Bacterial DNA was extracted from *Salmonella* isolates cultured overnight in Luria–Bertani broth using the MiniBEST Bacteria Genomic DNA Extraction Kit Ver.3.0 (Takara, Beijing, China), according to the manufacturer’s instructions. Eighteen genes known to be associated with resistance to the antibiotics as well as Class I integrons were tested by PCR using the oligonucleotide primers listed in Table 1. The PCRs were carried out using a MyCyler Thermocycler (BioRad, Hercules, CA, United States) in a 25 μL mixture that contained 0.5 mM of each primer, 1 × PCR buffer (Takara), 250 μM of dNTP, 0.5 U of Taq DNA polymerase (Takara), 1.5 mM MgCl_2_, and 0.5 μL of template DNA. The cycling conditions were 94°C for 5 min, followed by 30 cycles of 94°C for 30 s, 54–61°C for 30 s and 72°C for 1 min, and a final extension of 72°C for 10 min. PCR amplification products were separated on agarose gels and the DNA was purified from agarose plugs using the Takara Agarose Gel DNA Purification Kit (Takara). BGI Tech Solutions (Wuhan, China) performed DNA sequencing, and this data was aligned and analyzed using the Basic Local Alignment Search Tool (BLAST).

**TABLE 1:** Primer sequences used for gene screening.

**Resistance**			**Amplicon**	**Annealing**	
**gene type**	**Gene**	**Primer Sequence (5′ → 3′)**	**size (bp)**	**Temperature (°C)**	**References**
Chloramphenicol	*catA1*	F: GCAAGATGTGGCGTGTTACGGTGAA	258	56	This study
		R: TCATTAAGCATTCTGCCGACATGGA			
	*floR*	F: AACCCGCCCTCTGGATCAAGTCAA	549	56	Ghoddusi et al., 2015
		R: CAAATCACGGGCCACGCTGTATC			
	*cmlA*	F: ATTTTAGTTGGCGGTACTCCCT	307	61	This study
		R: CCCAGAAAGACTTCAGCCGAT			
Tetracycline	*tetA*	F: GCTACATCCTGCTTGCCTTC	210	56	This study
		R: CATAGATCGCCGTGAAGAGG			
	*tetB*	F: TTGGTTAGGGGCAAGTTTTG	659	56	This study
		R: GTAATGGGCCAATAACACCG			
	*tetC*	F: AAAGGCTATCAAGCTGTTTC	311	56	This study
		R: CCAGTAATGTATTCAAGGCTAA			
Sulfonamide	*sul1*	F: CTTCGATGAGAGCCGGCGGC	437	55	Aarestrup et al., 2003
		R: GCAAGGCGGAAACCCGCGCC			
	*sul2*	F: GCGCTCAAGGCAGATGGCATT	285	55	Aarestrup et al., 2003
		R:GCGTTTGATACCGGCACCCGT			
	*sul3*	F: AGATGTGATTGATTTGGGAGC	443	55	Zhang et al., 2009
		R: TAGTTGTTTCTGGATTAGAGCCT			
Quinolone	*qnrA*	F: AGAGGATTTCTCACGCCAGG	580	60	Cattoir et al., 2007
		R: TGCCAGGCACAGATCTTGAC			
	*qnrB*	F: GGMATHGAAATTCGCCACTG	264	56	Cattoir et al., 2007
		R: TTTGCYGYYCGCCAGTCGAA			
	*qnrS*	F: GCAAGTTCATTGAACAGGGT	428	57	Cattoir et al., 2007
		R: TCTAAACCGTCGAGTTCGGCG			
	*qnrD*	F: ACAGGAATAGCTTGGAAGGGTG	329	58	This study
		R: AAGATCGGAGCCACGAAACA			
β-lactamase	*bla*_TEM_	F: GAGTACTCACCATCACAGAAAAGC	489	58	Lu et al., 2010
		R: GACTTCCCGTCGTGTAGATAAC			
	*bla*_PSE_	F: AATGGCAATCAGCGCTTCCC	598	55	Shahada et al., 2006
		R: GGGGCTTGATGCTCACTACA			
	*bla*_CTX–M_	F: CGATGTGCAGCACCAGTAA	584	58	This study
		R: AGTGACCAGAATCAGCGG			
	*bla*_CMY–__2_	F: GACAGCCTCTTTCTCCACA	1015	57	This study
		R: TGGAACGAAGGCTACGTA			
	*bla*_SHV_	F: TTCGCCTGTGTATTATCTCC	807	60	This study
		R: TTTGCTGATTTCGCTCGG			
Class I integron	*qacE*Δ*1-F*	F: ATCGCAATAGTTGGCGAAGT	800	54	Zhou et al., 2014
	*sul1-B*	R: GCAAGGCGGAAACCCGCGCC			
	*intI1*	F: TGTCCACTGGGTTCGTGCCT	707	56	This study
		R: GCTTCGTGATGCCTGCTTGTT			
	5′-CS	F: GGCATCCAAGCAGCAAGC	Random	54.5	Meng et al., 2011
	3′-CS	R: AAGCAGACTTGACCTGAT			

### Conjugation Experiments

To determine whether resistance determinants were present in transferable genetic elements in *Salmonella* isolates, conjugation experiments were carried out using the broth mating method as previously described (Olsen et al., 2004). The *Salmonella* isolates positive for *bla*_CTX–M_ and/or integrons served as donors, and *E. coli* C600 was used as a recipient. Transconjugants were selected on Mueller–Hinton agar (Beijing Land Bridge Technology Co., Ltd.), containing 750 μg/ml rifampin and 100 μg/ml ampicillin. Susceptibility patterns of the transconjugants were determined by the methods described above. Co-transfer of resistance determinants was identified by amplifying the relevant genes from the transconjugants by PCR and determining the nucleotide sequencing of the PCR products. Plasmid DNA from transconjugants and donor strains was extracted utilizing a Tiangen Plasmid Purification Mini Kit (Tiangen Biotech, China) according to the manufacturer’s instructions. Replicon typing of plasmids was performed by a PCR-based method as previously described (Carattoli et al., 2005).

### MLST

Seven housekeeping genes (*aroC*, *dnaN*, *hemD*, *hisD*, *purE*, *sucA*, and *thrA*) were used for MLST as described online^1^. All PCR products were purified from agarose gels and sequenced by BGI Tech Solutions as described above. Alleles and STs were assigned according to the MLST scheme.

## Results and Discussion

### *Salmonella* Serotype Prevalence Among the Animal Products

Out of the 631 meat samples, 66 (10.5%) tested positive for *Salmonella*, and chicken had the highest prevalence (14.8%; 39/263), followed by duck meat (11.8%; 12/102), pork (8.8%; 7/80), and fish (4.3%; 8/186).

A total of 19 serotypes were identified among 66 *Salmonella* isolates and one isolate from chicken was un-typeable (Table 2). The 19 serotypes included 11 from chicken, and 8, 5, and 6 from duck meat, pork and fish, respectively. *S.* Thompson was the serotype most frequently identified (18.2%; 12/66), followed by *S.* Agona (13.6%; 9/66), *S.* Takoradi (12.1%; 8/66), and *S.* Typhimurium (10.6%; 7/66). *S.* Thompson was not isolated from pork but it was the most common serotype isolated from both duck meat and fish with 33.3% (4/12) and 37.5% (3/8), respectively. Although *S.* Thompson was the most prevalent serotype overall and chicken was the most likely to be contaminated with *Salmonella*, *S.* Thompson was not the most common serotype isolated from chicken. Both *S.* Agona and *S.* Takoradi were isolated from chicken at a higher rate (20.5%; 8/39 for each of the two serotypes) than S. Thompson (12.8%; 5/39) (Table 2).

**TABLE 2:** Distribution of *Salmonella* serotypes in retail meat.

**Serotype**	**Number (%) of isolates**				
	**Chicken**	**Duck**	**Pork**	**Fish**	**Total**
Thompson	5 (12.8)	4 (33.3)		3 (37.5)	12 (18.2)
Agona	8 (20.5)			1 (12.5)	9 (13.6)
Takoradi	8 (20.5)				8 (12.1)
Typhimurium	3 (7.7)	2 (16.7)	2 (28.6)		7 (10.6)
Enteritidis	4 (10.3)	1 (8.3)		1 (12.5)	6 (9.1)
Kentucky	5 (12.8)				5 (7.6)
Derby	1 (2.6)		2 (28.6)		3 (4.5)
Lindenburg	1 (2.6)	1 (8.3)		1 (12.5)	3 (4.5)
London	1 (2.6)		1 (14.3)		2 (3.0)
Anatum	1 (2.6)				1 (1.5)
Indiana	1 (2.6)				1 (1.5)
Galiema				1 (12.5)	1 (1.5)
Madelia				1 (12.5)	1 (1.5)
Paratyphi A			1 (14.3)		1 (1.5)
Senftenberg			1 (14.3)		1 (1.5)
Brunei		1 (8.3)			1 (1.5)
Brijbhumi		1 (8.3)			1 (1.5)
Muenchen		1 (8.3)			1 (1.5)
Rissen		1 (8.3)			1 (1.5)
Untypable	1 (2.6)				1 (1.5)
Total	39 (59.1)	12 (18.2)	7 (10.6)	8 (12.1)	66 (100)

*Salmonella* is a major cause of foodborne illness worldwide. In this study, *Salmonella* were recovered from retail raw meat products in Hubei Provence, China. The overall prevalence of *Salmonella* in retail meat products sampled in this study (10.5%) agreed with that reported in Henan Province, China (9.7%) (Yu et al., 2014) but was higher than that reported from Xinjiang, China (6.8%) (Yin et al., 2016). However, a simple conclusion could not be made from direct comparisons among studies due to regional differences, sampling seasons and the types of products sampled. In this study, the higher contamination rate found may be related to fewer biosecurity and hygiene measures inside these small markets.

We also identified 19 serotypes among the 66 isolates. The most common serotype in retail raw meat products was *S.* Thompson. While this result was consistent to two previous studies conducted in Iran (Soltan-Dallal et al., 2010; Sodagari et al., 2015), it differed from other reports in which the dominant serotype was *S.* Hadar in Xinjiang, China (Yin et al., 2016), *S.* Enteritidis in Henan Province, China (Yang et al., 2013) and *S.* Typhimurium in Brazil (Ristori et al., 2017). Other serotypes recovered in this study included *S.* Agona, *S.* Typhimurium, *S.* Enteritidis, *S.* Kentucky, and *S.* Derby, which have all been previously found in food products (Ma̧ka et al., 2014; Shah et al., 2017; Wang et al., 2017). The differences in dominant serotypes may be due to geographical differences, sample types or seasons and serotype pathogenicity.

### Antimicrobial Susceptibility

Among 66 *Salmonella* isolates, a high percentage of the isolates were resistant to sulfonamides (43.9%), tetracycline (43.9%), amoxicillin (36.4%), ampicillin (36.4%), chloramphenicol (27.3%), nalidixic acid (21.2%), streptomycin (18.2%), and cephazolin (15.2%) (Table 3). It was found that 17 (25.8%) of the 66 isolates were resistant to 1 antibiotic, 10 isolates (15.2%) were resistant to 2–3 antibiotics, 20 isolates (30.3%) were resistant to 4–6 antibiotics, 3 isolates (4.5%) were resistant to 7–9 antibiotics and a single isolate (1.5%) was resistant to 11 antibiotics (Table 4). Thus, 51 of the 66 *Salmonella* isolates (77.3%) were resistant to at least one antibiotic. *Salmonella* isolated from chicken were most likely to be resistant with 92.3% (36/39) of the isolates showing resistance to at least one antibiotic, and a single isolate exhibiting resistance to 11 antibiotics. *Salmonella* isolates from fish were the most susceptible to the antimicrobials tested with only 2 strains (25%) showing any resistance (Table 4).

**TABLE 3:** Antimicrobial resistance of *Salmonella* isolates from retail meat.

**Antimicrobial agent**	**Number of isolates (%)**				
	**Chicken**	**Duck**	**Pork**	**Fish**	**Total**
	**(*n* = 39)**	**(*n* = 12)**	**(*n* = 7)**	**(*n* = 8)**	**(*n* = 66)**
Sulfonamides	21 (53.8)	4 (33.3)	4 (57.1)		29 (43.9)
Tetracycline	20 (51.3)	3 (25)	6 (85.7)		29 (43.9)
Amoxicillin	16 (41.0)	4 (33.3)	3 (42.9)	1 (12.5)	24 (36.4)
Ampicillin	16 (41.0)	4 (33.3)	3 (42.9)	1 (12.5)	24 (36.4)
Chloramphenicol	15 (38.5)		3 (42.9)		18 (27.3)
Nalidixic acid	9 (23.1)	2 (16.7)	1 (14.3)	2 (25)	14 (21.2)
Streptomycin	10 (25.6)		2 (28.6)		12 (18.2)
Cephazolin	7 (17.9)	2 (16.7)	1 (14.3)		10 (15.2)
Gentamicin	3 (7.7)	1 (8.3)	1 (14.3)		5 (7.6)
Cefuroxime	2 (5.1)				2 (3.0)
Ciprofloxacin	1 (2.6)	1 (8.3)			2 (3.0)
Norfloxacin	1 (2.6)				1 (1.5)
Nitrofurantoin			1 (14.3)		1 (1.5)

**TABLE 4:** Multidrug resistance strains identified among *Salmonella* isolates recovered from retail meat.

**Sources**	**Number (%) of isolates resistant to indicated number of antimicrobials**					
	**1**	**2–3**	**4–6**	**7–9**	**11**	**Total (> 1)**
Chicken (*n* = 39)	11 (28.2)	8 (20.5)	14 (35.9)	2 (5.1)	1 (2.6)	36 (92.3)
Duck (*n* = 12)	3 (25)		4 (33.3)			7 (58.3)
Pork (*n* = 7)	2 (28.6)	1 (14.3)	2 (28.6)	1 (14.3)		6 (85.7)
fish (*n* = 8)	1 (12.5)	1 (12.5)				2 (25)
Total (*n* = 66)	17 (25.8)	10 (15.2)	20 (30.3)	3 (4.5)	1 (1.5)	51 (77.3)

The increasing frequency of antimicrobial resistance among *Salmonella* has become an emerging challenge to public health. Our results indicated that 30.3% of isolates were resistant to 4–6 antimicrobial agents and 4.5% were resistant to 7–9 antimicrobials. This indicated that antimicrobial resistance among *Salmonella* isolates from retail meat in Hubei Province was lower than that in other areas of China (Zhu et al., 2014; Wang et al., 2017). In our study, the highest rates of antimicrobial resistance were to the sulfonamides, tetracycline, amoxicillin, ampicillin and chloramphenicol. These results were consistent to studies from other regions in China and other countries (Ma̧ka et al., 2014; Yu et al., 2014; Abdi et al., 2017). The reasons for this may lie in the continuous and extensive use of these antimicrobials in food animals for disease treatment and growth promotion.

Cephalosporins and fluoroquinolones have been effectively used for the treatment of invasive and systemic salmonellosis in humans and animals (Bhan et al., 2005). Our study revealed that 15.2 and 3.0% of *Salmonella* isolates were resistant to first-generation (cephazolin) and second-generation cephalosporins (cefuroxime), respectively, and two isolates showed intermediate resistance to third-generation cephalosporins (cefotaxime) (Table 5). Previous work has described a decreasing susceptibility of *Salmonella* from retail foods to these antimicrobials (Hindermann et al., 2017; Kim et al., 2017). Interestingly, our results indicated that a high prevalence of cephazolin resistance was observed in isolates of *S.* Thompson, the predominant serotype. In this study, *Salmonella* resistance to nalidixic acid (21.2%) was not very common, however, 31.8% of the isolates also showed intermediate resistance to nalidixic acid (Table 5). Only 3.0 and 1.5% of the isolates were resistant to ciprofloxacin and norfloxacin, respectively. These rates were lower than those reported in other studies from China (Pan et al., 2009; Yin et al., 2016).

**TABLE 5:** Resistance phenotypes and antibiotic resistance genes from *Salmonella* isolates recovered from retail meat.

**Strain**			**Sequence**		**Intermediate**	
**no.**	**Source**	**Serotype**	**Type**	**Resistance phenotype**	**phenotype**	**Integrons/resistance genes**
1	Chicken	Not detected	463	AML, AMP, C, NA, TE		**Class I,** *qnrS, sul2, tetA*
2	Chicken	*S.* Agona	13		ST	*bla*_TEM–1_, *sul1*
3	Chicken	*S.* Agona	13	AML, AMP, ST, S3, TE		*bla*_TEM–1_,*sul3, tetA*
4	Chicken	*S.* Agona	13	AML, AMP, ST, S3, TE	NA	*bla*_TEM–1_, *qnrS, sul3, tetA*
5	Chicken	*S.* Agona	13	AML, AMP, ST, S3, TE	NA	*bla*_TEM–1_, *qnrS,sul3,tetA, floR*
6	Chicken	*S.* Agona	13	C	NA, ST	*qnrS, floR*
7	Chicken	*S.* Agona	13	AML, AMP, NA		*bla*_TEM–1_, *qnrB, sul1, sul2*
8	Chicken	*S.* Agona	1215	C	NA, ST	*qnrS, floR*
9	Chicken	*S.* Agona	1328	C	NA,ST	*qnrS, floR*
10	Chicken	*S.* Anatum	516	NA		*bla*_TEM–1_, *bla*_PSE–__1_, *sul1*
11	Chicken	*S.* Derby	20	S3, TE	NA, ST	*bla*_TEM–1_,*sul1,sul2,tetA*
12	Chicken	*S.* Enteritidis	11	AML, AMP, NA, ST, S3		**Class I,** *bla*_TEM–1_, *sul1*
13	Chicken	*S.* Enteritidis	11	AML, AMP, NA		**Class I,** *qnrB, qnrS, sul1, sul2, tetA*
14	Chicken	*S.* Enteritidis	11	AML, AMP, NA		**Class I,** *bla*_TEM–1_, *qnrB, sul1, sul2*
15	Chicken	*S.* Enteritidis	11	NA		*bla*_TEM–1_, *sul1*
16	Chicken	*S.* Indiana	17	AML, AMP, CXM, KZ, C, CIP, NA, NOR, ST, CN, S3	CTX, TE	*bla*_TEM–1_, *bla*_CTX–M–65_, *sul1, sul2, tetA*
17	Chicken	*S.* Kentucky	314	C, S3, TE	NA, ST	**Class I,** *qnrB, sul2, tetA*
18	Chicken	*S.* Kentucky	314	C, S3, TE	NA, ST	*qnrB, sul3, tetA*
19	Chicken	*S.* Kentucky	314	C, S3, TE	NA, ST	**Class I,** *qnrB, sul4, tetA*
20	Chicken	*S.* Kentucky	314		ST	
21	Chicken	*S.* Kentucky	314		ST	*floR*
22	Chicken	*S.* Lindenburg	46	S3		*bla*_TEM–1_, *sul1*
23	Chicken	*S.* London	155	AML, AMP, C, S, CN, S3, TE		**Class I,** *bla*_TEM–1_, *qnrB, sul1,floR*,
24	Chicken	*S.* Takoradi	541	TE	NA, ST	*qnrS, tetA*
25	Chicken	*S.* Takoradi	541	TE	NA	*qnrS, tetA*
26	Chicken	*S.* Takoradi	541	C, ST, S3, TE		*sul2, tetA*
27	Chicken	*S.* Takoradi	541	TE	NA	*qnrS, tetA*
28	Chicken	*S.* Takoradi	541	C, ST, S3, TE	NA	*qnrS, sul2,tetA, floR*,
29	Chicken	*S.* Takoradi	541	C, ST, S3, TE	NA	*sul2,tetA, floR*
30	Chicken	*S.* Takoradi	541	C, ST, S3, TE		*sul2,tetA,floR*
31	Chicken	*S.* Takoradi	541	C, ST, S3, TE		*floR, sul3, tetA*
32	Chicken	*S.* Thompson	26	AML, AMP, KZ, S3, TE	CXM, NA, ST	**Class I,** *bla*_TEM–1_, *qnrB, sul1, tetA*
33	Chicken	*S.* Thompson	26	AML, AMP, KZ, S3	CXM, NA	*qnrB, tetA*
34	Chicken	*S.* Thompson	26	AML, AMP, CXM, KZ, C, CN, S3, TE	CTX, ST	**Class I: *dfr17-aadA5*,** *bla*_TEM–1_, *bla*_CTX–M–15_, *qnrB, sul1*
35	Chicken	*S.* Thompson	26	AML, AMP, KZ, S3	ST	**Class I,** *bla*_TEM–1_, *sul1*
36	Chicken	*S.* Thompson	26	AML, AMP, KZ, S3	ST	**Class I,** *sul1*
37	Chicken	*S.* Typhimurium	19	NA		*bla*_TEM–1_, *sul1*
38	Chicken	*S.* Typhimurium	1682	KZ	AML	*bla*_TEM–1_
39	Chicken	*S.* Typhimurium	1682	AML, AMP, TE	S3	*bla_TEM–1_,sul1*
40	Duck	*S.* Braenderup	22	NA		*bla*_TEM–1_
41	Duck	*S.* Brunei	2256			
42	Duck	*S.* Enteritidis	11	AML, AMP, CIP, CN		**Class I,** *sul1*
43	Duck	*S.* Lindenburg	46	S3		*bla*_TEM–1_, *sul1*
44	Duck	*S.* Muenchen	82			
45	Duck	*S.* Rissen	469	AML, AMP, S3, TE		*sul1, sul3, tetA*
46	Duck	*S.* Thompson	26		ST	*bla*_TEM–1_, *sul1*
47	Duck	*S.* Thompson	26	AML, AMP, KZ, S3, TE	NA	**Class I: *dfrA12-aadA2*,** *qnrB, sul1, tetA*
48	Duck	*S.* Thompson	26	AML, AMP, KZ, S3, TE	CXM, NA, ST	**Class I,** *bla*_TEM–1_, *qnrB, sul1, tetA*
49	Duck	*S.* Thompson	26			
50	Duck	*S.* Typhimurium	19	NA		*bla*_TEM–1_, *sul1*
51	Duck	*S.* Typhimurium	99		ST	*bla*_TEM–1_, *sul1*
52	Pork	*S.* Derby	40	TE	C, NA, S3	*sul1, tetA*
53	Pork	*S.* Derby	40	TE	C, NA	*sul1, sul2, tetA*
54	Pork	*S.* London	155	AML, AMP, C, ST, S3, TE	NA	**Class I: *aacA4C- arr-3- dfr2*,** *bla_TEM–1_, qnrB, sul1, sul2, tetA*
55	Pork	*S.* ParatyphiA	463	KZ, S3, TE		*sul1, tetC*
56	Pork	*S.* Senftenberg	14			
56	Pork	*S.* Typhimurium	313	AML, AMP, C, ST, S3, TE		**Class I:*aadA2*,** *bla*_TEM–1_, *qnrB, sul1, sul2*
58	Pork	*S.* Typhimurium	19	AML, AMP, C, NA, CN, F, S3, TE	CIP, NOR, ST	**Class I:*dfrA12-aadA21*,** *bla_TEM–1_, sul1, tetB, tetC*
59	fish	*S.* Agona	13			
60	fish	*S.* Enteritidis	11	AML, AMP, NA		**Class I,** *bla*_TEM–1_, *qnrB, sul1*
61	fish	*S.* Galiema	501		ST	*bla*_TEM–1_, *sul1*
62	fish	*S.* Lindenburg	45		S3	*bla*_TEM–1_, *sul1*
63	fish	*S.* Madelia	226	NA		*bla*_TEM–1_, *sul1*
64	fish	*S.* Thompson	26			
65	fish	*S.* Thompson	26			
66	fish	*S.* Thompson	26			

**Class I, class I integrons which did not possess antibiotic resistance gene cassettes; AML, Amoxycillin; AMP, Ampicillin; KZ, Cephazolin; CXM, Cefuroxime sodium; CTX, Cefotaxime; C, Chloramphenicol; NA, Nalidixic acid; NOR, Norfloxacin; CIP, Ciprofloxacin; ST, Streptomycin; CN, Gentamicin; F, Nitrofurantoin; S3, Sulfonamides compound; TE, Tetracycline.**

### The Occurrence of Antimicrobial Resistance Genes and Class I Integrons

All 66 *Salmonella* isolates were screened for the presence of antimicrobial resistance genes and class 1 integrons. Numerous oligonucleotide primer pairs (Table 1) were used to screen the 66 *Salmonella* isolates by PCR for the presence of genes encoding resistance to chloramphenicol, tetracycline, sulfonamides, quinolones, and β-lactam antibiotics. Overall, the results showed a strong correlation between antimicrobial resistance genotypes and phenotypes (Table 5).

Among 26 β-lactam-resistant isolates in this study, four different β-lactamase genes were identified, including *bla*_TEM–1_, *bla*_CTX–M–65_, *bla*_CTX–M–15_, and *bla*_PSE__–__1_ (Table 5). The *bla*_TEM–1_ gene had the highest occurrence (69.2%, 18/26) among β-lactam-resistant isolates; a rate similar to that previously reported for *Salmonella* isolated from meat and dairy products in Egypt (Ahmed et al., 2014). The extended-spectrum β-lactamase (ESBL) genes, *bla*_CTX–M–65_ and *bla*_CTX–M–15_, were each observed in a single *S.* Indiana and *S.* Thompson isolate, respectively. The *bla*_PSE–__1_ gene was also identified in only one isolate of *S.* Anatum. A cephalosporin resistant *S.* Thompson isolate carried the gene encoding CTX-M-15, which is the most prevalent ESBL worldwide (Chon et al., 2015). In this study, the rarely observed *bla*_CTX–M–65_ was present in a ciprofloxacin- and cefuroxime-resistant isolate of *S.* Indiana. Similarly, it was reported that the *bla*_CTX–M–65_ gene was harbored in ciprofloxacin- and cefotaxime-resistant *S.* Indiana isolates in Henan Province of China (Bai et al., 2016). In addition, the *bla*_CTX–M–65_ gene was identified previously in an outbreak of *S.* Infantis in Ecuador (Cartelle Gestal et al., 2016).

The *qnr* family of genes encodes a pentapeptide repeat protein that protects DNA gyrase and topoisomerase IV from quinolone and fluoroquinolone inactivation. Plasmids containing *qnr* can be transferred among bacteria *via* conjugation and this is a very important emerging public health concern (García-Fernández et al., 2009). Although *qnr* confers only low-level quinolone resistance, mutations in the quinolone resistance-determining region (QRDR) could result in higher-level resistance to fluoroquinolones, especially to ciprofloxacin. The existence of *qnr* genes has been reported in *Salmonella* from other regions in China and in other countries (Yang et al., 2013; Wasyl et al., 2014). In the current study, 62.9% (22/35) of the 14 nalidixic acid-resistant and 21 intermediate resistant *Salmonella* isolates harbored *qnrB* and/or *qnrS* genes (Table 5). The *qnrB* gene was present in 34.3% (12/35) of the nalidixic acid resistant or intermediate resistant *Salmonella* isolates while *qnrS* was present in 31.4% (11/35) of the isolates. Both *qnrB* and *qnrS* were found in only a single isolate of *S.* Enteritidis. The genes *qnrA* and *qnrD* were not found among any of the isolates. The *bla*_CTX–M–15_ gene was found in combination with *qnrB*, while the isolate with *bla*_CTX–M–65_ carried no quinolone resistance genes.

The tetracycline resistance genes (*tetA*, *tetB*, and *tetC*) and sulfonamide resistance genes (*sul1*, *sul2*, and *sul3*) were identified in 86.7 and 96.9% of the strains resistant or intermediate resistant to the cognate antibiotic. The *floR* gene was identified in 40% (8/20) of chloramphenicol-resistant isolates (*n* = 18) and intermediate resistant isolates (*n* = 2) (Table 5).

An integron is a mobile DNA element, which usually contains one or more antimicrobial resistance genes that can be transferred among bacteria. In this study, Class I integrons (intI1) were found in 18 isolates (27.3%) and 13 of them did not possess antimicrobial resistance gene cassettes. The remaining 5 intI1-positive isolates contained five groups of resistance gene cassettes including *aacA4C-arr-3-dfr2*, *dfrA12-aadA21*, *aadA2*, *dfrA12-aadA2*, and *dfr17-aadA5* (Table 5). Our study also indicated that all of the class I integron-positive isolates were resistant to at least three classes of antimicrobials. This supports the hypothesis of the association between class I integrons and MDR in *Salmonella* isolates (Abraham et al., 2016; Meng et al., 2016).

Previous investigators identified class I integrons in foodborne *Salmonella* isolated in China at rates that ranged from 20 to 61% (Zhu et al., 2014; Meng et al., 2016; Li et al., 2017). In this study, the gene cassettes *aac*A*4C-arr-3-dfr2* from *S.* London exhibited >98.0% nucleotide sequence identity to that of the variable region of Integron In1021 from a clinical *E. coli* isolated in China (accession no. KR338349), suggesting that some of its components may have originated from other species of Enterobacteriaceae such as *Escherichia*. The existence of class I integrons containing genes for antimicrobial resistance in *Salmonella* isolates poses a threat to public health and food safety.

### Transferability of bla_CTX–M_ Genes and Integrons

Six *Salmonella* strains harboring the ESBL *bla*_CTX–M_ gene and integrons were selected as donor strains in conjugation experiments. These donor strains were mated with an *E. coli* C600 recipient strain. Four transconjugants were obtained and characterized. PCR based replicon typing revealed that plasmids belonging to incompatibility types H12 and N were transferred from *Salmonella* to the *E. coli* C600 (Table 6). However, an incA/C plasmid in a *S*. Indiana isolate was not transferred. Genes present in the transconjugants included class I integrons, *bla*_TEM–1_, *bla*_CTX–M–65_, *bla*_CTX–M–15_, *qnrB, sul1, sul2*, *tetA*, and *tetB*. The co-transfer of *bla*_CTX–M–15_ and *qnrB* was observed with an integron from an *S*. Thompson isolate to *E. coli* strain C600 (Table 6). When mated with the *S.* London donor, the *E. coli* C600 transconjugants showed an identical antimicrobial resistance phenotype to the donor. For the other *Salmonella* donors, the *E. coli* C600 recipients showed similar, but not identical antimicrobial resistances (Table 6).

**TABLE 6:** Characteristics of *Salmonella* isolates and *E. coli* transconjugants.

**Isolate**				**Replicon**
**No.**	**Serotype**	**Resistance profile**	**Resistance genes**	**typing**
16	*S.* Indiana	AML, AMP, CXM, KZ, C, CIP, NA, NOR, ST, CN, S3	*bla*_TEM–1_, *bla*_CTX–M–65_, *sul1*, *sul2*, *tetA*	H12, A/C
16-T		AML, AMP, CXM, KZ, C, CIP, NA, NOR, ST, CN,	*bla*_TEM–1_, *bla*_CTX–M–65_, *tetA*	H12
34	*S.* Thompson	AML, AMP, CXM, KZ, C, CN, S3, TE	Class 1: *dfr17-aadA5, bla*_TEM–1_, *bla*_CTX–M–15_, *qnrB, sul1*	H12
34-T		AML, AMP, CXM, KZ, CN	Class 1: *dfr17-aadA5, bla*_TEM–1_, *bla*_CTX–M–15_, *qnrB*	H12
54	*S.* London	AML, AMP, C, ST, S3, TE	Class 1: *aacA4C-arr-3-dfr2*, *bla*_TEM–1_, *qnrB*, *sul1*, *sul2*, *tetA*	N
54-T		AML, AMP, C, ST, S3, TE	Class 1:*aacA4C-arr-3-dfr2*, *bla*_TEM–1_, *qnrB*, *sul1*, *tetA*	N
58	*S.* Typhimurium	AML, AMP, C, NA, CN, F, S3, TE	Class 1:*dfrA12-aadA21, bla*_TEM–1_, *sul1*, *tetB*, *tetC*	H12
58-T		AML, AMP, C, NA, CN, F, TE	Class 1:*dfrA12-aadA21, bla*_TEM–1_, *tetB*	H12

**AML, Amoxycillin; AMP, Ampicillin; KZ, Cephazolin; CXM, Cefuroxime sodium; C, Chloramphenicol; NA, Nalidixic acid; NOR, Norfloxacin; CIP, Ciprofloxacin; ST, Streptomycin; CN, Gentamicin; F, Nitrofurantoin; S3, Sulfonamides compound; TE, Tetracycline.**

Of concern, the findings of conjugation tests demonstrated the presence of genes including *bla*_TEM–1_, *bla*_CTX–M–15_, and *qnrB* on mobile plasmids. These resistance genes and integron were conjugally transferred from a *Salmonella* donor strain and conferred a MDR phenotype to a naïve *E. coli* recipient. Few studies have shown the co-existence of integron with plasmid-mediated quinolone resistance (PMQR) determinants and ESBL genes on transferable plasmids (Lai et al., 2013). It has been suggested that the transmissible elements containing these genes could be selected and transmitted during exposure to any of the antimicrobials (González and Araque, 2013; Jiang et al., 2014).

### MLST Analysis

Thirty-two STs were identified among the 66 isolates, and ST26 (*n* = 12) was the most common followed by ST541 (*n* = 8), ST13 (*n* = 7), ST11 (*n* = 6), and ST314 (*n* = 5) (Table 5). The STs in this study were correlated with specific serotypes, such as ST11 with *S.* Enteritidis, ST13 with *S.* Agona, ST19 with *S.* Typhimurium, ST314 with *S.* Kentucky and ST26 with *S.* Thompson. Though a particular ST was always associated with the same serotype, some serotypes could be represented by multiple STs. For example, the isolates characterized as *S*. Derby belonged to ST20 and ST40.

The diversity of samples we collected was also reflected in a diversity of the 32 STs that we identified. ST26 was the most common especially among poultry samples and this ST corresponded to *S.* Thompson that has a low rate of occurrence in China (Ni et al., 2018). ST11 and ST19 were the most frequent sequence types recovered from meat samples and these STs are commonly associated with human salmonellosis outbreaks (Gunel et al., 2015; Ashton et al., 2016). Additionally, our results revealed that the serotypes and STs were highly correlated, which is consistent with previous research (Achtman et al., 2012). We did find sequence type differentiation within serotypes such as with *S.* Typhimurium and *S.* Derby. Therefore, MLST analysis provided a greater discriminatory power than serotype determination.

## Conclusion

This study identified *S.* Thompson and *S*. Agona as the most common serotypes among *Salmonella* isolates recovered from retail raw meat products in Hubei Province, China. Antimicrobial resistance was prevalent and correlated well with the presence of cognate resistance genes. *Salmonella* isolates containing class I integrons showed broader antimicrobial resistance than those without them. In addition, conjugation experiments revealed a co-transfer of class I integron with PMQR and ESBL genes. The reasonable use of antibiotics in livestock breeding and management of the food supply chain is of importance to ensure food safety.

## Data Availability Statement

Publicly available datasets were analyzed in this study. This data can be found here: http://enterobase.warwick.ac.uk/species/senterica/allele_st_search.

## Author Contributions

XS and HW designed the study and revised the manuscript. MZ conducted the experiments and interpreted the results. XL and WH assisted in the completion of the experiments. GP rewrote the discussion and revised the manuscript.

## Conflict of Interest

The authors declare that the research was conducted in the absence of any commercial or financial relationships that could be construed as a potential conflict of interest.

## Footnotes

1 http://mlst.warwick.ac.uk/mlst/dbs/Senterica
